# What's "quickest and easiest?": parental decision making about school trip mode

**DOI:** 10.1186/1479-5868-7-62

**Published:** 2010-08-06

**Authors:** Guy EJ Faulkner, Vanessa Richichi, Ronald N Buliung, Caroline Fusco, Fiona Moola

**Affiliations:** 1 University of Toronto, Faculty of Physical Education and Health, University of Toronto, 55 Harbord Street, Toronto, ON, M5 S 2W6, Canada; 2 Department of Geography, University of Toronto at Mississauga, 3359 Mississauga Road N, South Building, Mississauga, ON, L5L 1C6, Canada

## Abstract

**Background:**

The potential benefits of active school travel (AST) are widely recognized, yet there is consistent evidence of a systematic decline in the use of active modes of transportation to school since the middle part of the 20^th ^century. This study explored parental accounts of the school travel mode choice decision-making process.

**Methods:**

Thirty-seven parents of children (17 who walked; 20 who were driven) from four elementary schools in Toronto, Canada participated in semi-structured interviews. The schools varied with respect to walkability of the built environment and socio-economic status. Thematic analysis of interview transcripts identified a two-stage decision-making process.

**Results:**

An initial decision concerned the issue of escorting or chauffeuring a child to/from school. This decision appeared to be primarily influenced by concerns about traffic, the child's personal safety, and the child's maturity and cognitive ability regarding navigating his/her way to/from school safely. Following the escort decision, parents considered mode choice, typically selecting what they perceived to be the easiest and most convenient way to travel. The ascription of convenience to the various modes of transportation was influenced by perceptions of travel time and/or distance to/from school. Convenience became a particularly salient theme for parents who found it necessary to complete multi-activity trip chains.

**Conclusions:**

The school travel mode choice decision process is complex. Future research and practice should continue to address safety concerns that are typically the focus of active school transport initiatives while addressing more explicitly the behavioural cost of competing mode choices.

## Background

In Canada, the percentage of children classified as overweight or obese rose over the last twenty years from 14% to 31% among boys, and from 14% to 25% among girls [1]. The causes of overweight and obesity, and the potential solutions to preventing and reducing obesity prevalence, are complex. We live in obesogenic environments that increasingly promote high energy intake and physical inactivity [2]. Spanier, Marshall and Faulkner [3] argue that this obesity pandemic is not just a matter of decreased physical activity levels but is partly influenced by increased involvement in sedentary behaviours. One example is the consistent decline in the use of active modes (i.e., walking, biking) to and from school observed in Western nations (see [4] for a review). In the Greater Toronto Area, Canada's largest city-region, walking mode share for trips to school declined between 1986 and 2001 (53% - 42% for 11-13 year olds, 39% - 31% for 14-15 year olds) [5]. Arresting this decline would not only reduce time engaged in a sedentary behaviour (passive commuting by car), it would also replace such sedentary behaviour with moderate intensity physical activity (active commuting by walking). How best to encourage this type of behavioural shift or what decision-making processes are involved remain unclear.

McMillan [6] developed the first conceptual framework to highlight factors that may influence parents' decisions about how elementary school children travel to school. In a recently proposed framework to help researchers organize future studies of active school transport [AST; Ecological and Cognitive Active Commuting (ECAC) framework], Sirard and Slater [4] identify different levels of influence at policy, neighbourhood and parent/family levels. As with McMillan's framework [6], parents are assumed to make the ultimate decision about whether their child can walk to school or not. The decision may be influenced by perceptions of the physical and social environments which combine with attitudes, beliefs, and perceptions of social norms about their child using AST. An extensive array of correlates of AST have been identified and can be integrated within this framework including demographic, individual and family factors, school factors, and social and physical environmental factors (see [4] for a review).

In reviewing the broader literature and recent reviews [4,6-9], one key knowledge gap remains. The majority of existing research has adopted a cross-sectional, survey methodology. Such an approach is important in identifying the factors associated with a given transport mode but does not lend itself to exploring the dynamic nature of the actual decision making processes underlying the transport mode choice. Authors of recent reviews acknowledge that "the question of what determines the travel behaviour for the trip to school has yet to be answered" [6]. Indeed research to date fails to "consider the potentially complex role parents' decision making play in controlling their children's travel behaviours and how environmental characteristics interact with these processes" [8]. Qualitative approaches may be particularly helpful for unravelling the complexities of travel behaviour [10].

At least five qualitative studies on school travel behaviour have been published [11-15]. These have largely explored the perceived benefits and barriers associated with active school travel. This research has revealed important perceived barriers to AST, such as inconvenience, inclement weather, and safety concerns. For example, the impact of parents' work schedules on children's active transport choices may be magnified during "chaotic" mornings [14]. In addition, negative perceptions of neighbourhood safety such as fear of child abduction, neighbourhood violence, traffic volume and speed, the influence of media "kidnapping" stories, and not trusting children's 'under-developed' judgments, may deter parents from allowing children to walk to school [11,14].

While informative, these studies do not explicitly untangle and match up the school travel decision making process with the reported barriers and facilitators. Additionally, these studies do not explore travel mode choice by sampling parents from different geographic areas. That is, no controls were placed on differences across built environments. McMillan [6] and Panter and colleagues [8] highlight the importance of urban form and sociodemographics in the travel mode choice decision making process. This study addresses these concerns through a qualitative investigation of the parental decision making process that gives rise to a child's use (autonomously or otherwise) of a particular transport mode for journies to and from school. This decision-making process was explored among parents whose children went to schools differing with respect to neighbourhood socioeconomic status (low versus high) and built environment (i.e., period of development and street layout) across the Greater Toronto Area (GTA), Canada's largest and most culturally diverse metropolitan region.

## Methods

### Sample

Ethics approval for the study was granted by institutional ethics boards. A sample of four elementary schools in the GTA was recruited to capture diversity with respect to neighbourhood built environment characteristics and socioeconomic status (SES). The rationale for sampling from heterogeneously designed environments is that the structure of the transportation system (e.g., road layout), and geographical organization of buildings is conceptualized as a determinant of pedestrian behaviour. Elementary schools within Toronto's inner suburbs (e.g., typically characterized as having curvilinear, looping streets, with less pedestrian connectivity and walkability) that border the city centre, and schools from within the traditional downtown urban core (i.e., characterized by gridded streets, with higher levels of pedestrian connectivity and walkability) were chosen. Moreover, given the consistent finding that AST is related to household income [16-18], school selection also involved an examination of school location against median household income (from the 2001 Canadian Census) in and around the immediate vicinity of each school. The following elementary schools within one school board were recruited based on neighbourhood design and SES: School D (grid streets - low SES); School B (grid streets - high SES); School T (looping streets - low SES); and School R (looping streets - high SES).

Five AST dyads and five non-AST dyads were the recruitment target for each school. However, only eight parents were recruited from School T, and one guardian from School D did not complete the interview. Therefore, data were collected from a total of 37 parents - seventeen with a child who walked to/from school, and 20 with a child who was driven. The mean age of the children was 9.8 years. All of the participants (with the exception of one guardian who did not take part in an interview) were parents; more mothers (n = 30) than fathers (n = 7) participated and the average age of the sample was 40.3 years. AST children (all walkers) lived, on average, 558 metres (SD 192 m) from their schools. Children who were driven lived approximately 3.2 km (SD 3280) from the school, although twelve of these participants lived within 1.6 km from school. Within this school board, children living further than 1.6 km from school are eligible for a school bus service. Schools situated within the low SES neighbourhoods had a high population of students/parents whose first language was not English; interpreters (1 Cantonese and 2 Vietnamese) were used for three interviews. Further descriptive information about the schools is provided in Table 1.

**Table 1:** School Profiles

	School D	School B	School T	School R
Neighborhood	Gridded streets	Gridded streets	Looping streets	Looping streets
Socioeconomic Status	Low	High	Low	High
Number of students	289	398	403	240
Primary language other than English	180	29	234	130
Grade Levels	JK to Grade 5	JK to Grade 6	JK to Grade 5	JK to Grade 6
Recommended student arrival	8.30 -8.40 a.m.	None	Not before 8.30 a.m.	Not before 8.30 a.m.
Existing School Travel Initiatives	Walk to School Week	None	Walk to School Day	Walking School Bus Scheme

### Data Collection

A semi-structured interview was conducted with each parent. The purpose of interviewing is to allow researchers to enter into the other person's perspective and to create knowledge through the interaction between the interviewer and the interviewee [19].

### Theoretical Framework

The interview guide was informed by perspectives from behavioural economics (see [20]). Raynor, Coleman, and Epstein [21] suggest that if we conceptualize activity patterns as a series of choices between being physically active (AST) and sedentary (NON AST), behavioral economics can help us to understand the factors that influence how we allocate our time. Unlike rational choice theories, BE posits that decision-making is not just about weighing the pros and cons of each alternative, but is a response to the circumstances of a given situation. Epstein [20] outlines four general principles of BE. First, the choice and reinforcing value of an activity depends on what alternatives are available. For example, the main reason a parent/guardian may not drive his or her child to/from school may be due to a lack of access to a vehicle. In this case, active modes of transportation may be the only feasible option. Second, the choice of an alternative depends on the behavioural cost, or work needed to access an activity. One way to reduce sedentary behaviours would be to increase the cost of choosing to be sedentary and increase the accessibility (or convenience) of active alternatives. Within a travel mode choice context, for example, restricted parking and highly connected walking/cycling paths within school neighbourhoods may increase AST participation. Third, choices are based on the reinforcing value (or reward) of engaging in a behaviour. For example, some people may enjoy driving to school rather than walking, or they may continue to drive to school because it allows them to get their children to school on time, or enables the parent/guardian to travel to a next activity in a timely fashion (e.g., work, shop, etc.). Fourth, choices depend on the delay (or immediacy) between choosing and receiving the alternative or reinforcer. Individuals may assign less importance to outcomes in the distant future than those in the present. Accordingly, questions in the interview schedule were asked about what travel modes were available to get to/from school; what was the behavioral cost of each mode; what reinforced travel mode decisions; and how decisions were made. Examples of questions are included in Appendix 1. Interviews lasted between 45 minutes and an hour.

### Data Analysis

The audio-taped interviews were transcribed verbatim and, using thematic analysis, the data were categorised into themes. Identifying, analyzing, and reporting patterns in the data is a standard method for organising and describing a qualitative data set in detail [22]. The analysis process was both inductive and iterative. Interview transcripts were read line by line and sentences, phrases, and clauses were each assigned a code, remaining close to children's words. Using the constant comparison method, each transcript was initially read and searched for commonly occurring emergent units of meaning [23]. These units of meaning were carefully coded and contrasted against other emerging units of meaning. The data were then read in their entirety; this allowed for the confirmation of existing meaning units and the generation of new ones. Commonly occurring patterns of meaning across all participants' narratives were grouped together into categories. After refining main themes, the data were searched for the particular subcategories that give rise to themes, as well as broad inter-relationships between themes [23,24]. Specifically, the parents described how the school travel mode was informed by two separate decisions. The first decision concerned whether their child needed escorting to school. The second decision concerned the mode choice which was informed by perceptions of the behavioural cost of alternative transport modes. These two decisions form the basis of the results section (see Appendix 2 for a description of transcription codes). The identification of these themes was reached through consensus among three members of the research team.

## Results

### Travel Mode Choice

Parents typically described themselves as the ultimate decision makers when it came to how their children travelled to/from school: "My husband and I [decide] ... because we're the parents and we're looking after them so we have to find the best ways to send them to school" (RP8NON). Rather than engaging in any type of negotiation with the child regarding travel mode, parents did not offer any alternatives: "[T]hey've never really had a choice, you know, we've always walked and that's the way it is" (BP5AST).

Although participants described many factors that influenced their travel mode choices, they discussed school travel as a habitual behaviour, a "routine" (TP7NON) involving "no real thought" (BP9NON) because it was something they did on a daily basis: "I suppose it's habitual because obviously it's what we do all the time" (RP2AST). One participant from School R described the trip to school as being as routine as waking up in the morning:

No [I don't think about it], if anything I don't even have that in my mind; it's like okay, we've got to go to school the same way - you have to stand up to wake up is the same way we have to take the car to school. (RP8NON)

Although these parents may have taken certain factors into consideration (see sections to follow) when they first started making mode choices for their children's trips to/from school, these initial decisions seemed to develop into routinized behaviour that no longer required a conscious decision-making process.

### The Trip to School: Two decisions

From a behavioural economics perspective, the option with the least behavioural cost for parents was allowing their child to travel independently to school. However, this was the option that parents had most difficulty with because many of them felt that it was not safe for their child to commute independently.

### Decision One - To Escort or Not to Escort?

NON AST parents from schools R and T (situated in neighbourhoods characterized by looping streets), in particular, expressed concerns about road safety, which resulted in their hesitation about allowing their children to travel independently to/from school. "Crossing a major intersection" (RP7NON) was a common concern for these parents, as one mother from School T explained:

I know she [my daughter] would look [both ways when crossing the street], yes, but I don't know, sometimes in a split of a second something can happen. Let's say I allow her to walk with a group of friends when she's twelve ... and they didn't see some vehicle coming ... and something happens. It's unpredictable for that. (TP7NON)

Parents were also frightened that their children "might meet a stranger on the way to school" (BP1AST). Both AST and NON AST parents voiced concerns about not knowing "who they'll sort of meet along the way" (BP7NON); many expressed concerns about their child meeting "some freak person," (BP4AST) or "some wacko grabbing her and putting her in the back of the truck" (BP6NON). One AST parent explained, "I don't think somebody would come up to them and talk to them and maybe take them away, but there's always that fear I guess" (RP3AST). NON AST parents from schools R and T (looping streets) only expressed concern about road safety as a motivation for potentially limiting their children's independent travel.

Both AST and NON AST parents also expressed discomfort about allowing their children to travel alone to/from school because they believed their children were "still too young" (BP7NON), "not ready" (BP10NON), or not "responsible enough to cross the street" (TP10NON). One AST parent explained the importance of having the maturity and skills to travel safely:

[I]t's a matter of wondering if she has the skill level yet to deal with situations that may occur ... Just recognizing a good scene or a bad scene or a potential for something that's not right occurring and being able to pull back from it and go that distance to say 'Hey, no, this isn't for me, I'm outta here.' (RP5AST)

Most parents however did comment that they might consider allowing their child to travel to school independently once they had reached the age of twelve, which would coincide with their child changing schools to enter grade seven (at approximately 12 years of age).

Parents also talked about that ways in which they had worked on overcoming the fears they had about their child's independent travel. Both AST and NON AST parents who have allowed their children to travel alone occasionally in the past spoke about how they stayed connected to their children in order to ensure their safety. Many parents talked about how they "spy on our kids" (BP2AST) to "watch them ... to see what they're doing" (TP2AST). One AST mother explained that when her children first started walking alone in the neighbourhood, she observed their behaviour by walking behind them:

I let them go [when my eldest was 10] and then I would walk behind them to see what they do on the street ... See what they're doing in terms of, like, if they were to start walking by themselves what would they be on the street doing. So I used to walk and watch them, but, they just walked. (TP2AST)

Another way parents ensured that their children arrived home safely was by maintaining communication via cellular telephone or land line. Some parents would "always let them [their children] have a phone" (DP10NON) so that they could call for emergency purposes, while others instructed their children to "call me when you get home" (DP1AST) "so that I know that you're home safely" (DP7NON).

Parents from all of the schools sampled also described travelling with groups of friends as a way of overcoming the fears and hesitations associated with their children travelling to/from school independently. Parents who occasionally allowed their children to travel to/from school without adult supervision ensured that "there's always that buddy system" (BP9NON) and that their children were travelling "with a group of friends" (TP7NON). Some parents explained that they would consider independent travel if there are other children to walk with or "if it's a whole bunch of kids walking up at the same time" because there is "safety in numbers" (BP1AST). One parent from School B commented, "if she [my child] wanted to walk with friends and it was safe then I would be okay with that" (BP7NON).

Parents also explained that they would feel safer about their children travelling to and from school when "there's [sic] so many people about" (RP2AST) and "there's lots of people on the road" (BP4AST). One mother from School B explained that her daughter "knows a lot of people on the way to school, so I know that if there was a problem, there would be people that she could go to their home and get help" (BP1AST). Knowing or being familiar with the people in the neighbourhood who will "kind of watch out" (DP6NON) for their children would provide a sense of comfort for these parents. "It's about social capital along the way too - do you know people, are you likely to run into somebody who will keep an eye out for him" (DP9NON).

For participants in this study, allowing their children to travel independently to school would automatically eliminate driving as a travel mode choice. However, since these parents, typically, escorted their child(ren) to school, the second step in the decision making process is whether to use active or inactive modes in escorting their child to/from school. In this study, the two competing modes were walking or driving and each was considered in terms of its behavioural cost.

### Decision Two - Walk or Drive?

#### Behavioural Cost

All parents stated that they would choose the most efficient mode to travel to/from school with their children. AST parents described their walk to/from school as "fairly fast" (DP2AST). One parent stated that compared to driving "it's faster to walk than get the car out of the driveway" (BP2AST). In addition, the lack of parking made walking to/from school the faster choice. Another parent from School D commented, "Even if we drove we'd still have to find parking and so it's basically an even balance. Walking is the fastest" (DP2AST). Issues of time were especially prevalent among the NON AST parents, who spoke more about their busy work schedules and having no time available to even consider walking as a possible alternative. Many explained, "if I had more time" (TP6NON) they would be more likely to walk to/from school more often. They discussed the weekday morning time crunch and explained that "basically what it takes to be able to walk in the morning is just leave 20 minutes earlier" (DP9NON). This would involve "just getting more disciplined and getter up a bit earlier" (DP9NON). Although waking up earlier was "feasible" (TP8NON), one parent laughed, "Do I want to wake up earlier? No!" (RP8NON).

Issues of time related to the trip to/from school were inextricably linked to distance/proximity. AST parents walked because of their proximity to the school: "We're lucky because, as I said, we're like a block and a half [from the school]" (BP1AST). For those who drove to school, distance was also an important influencing factor: "We're just unfortunate that we don't really have a choice because it's too far" (BP10NON). This perception was held among NON-AST parents who lived both within or beyond the distance (1.6 km) for school bus eligibility.

Both AST and NON AST parents described their respective travel modes as being "easy" (TP1AST) "simpler" (TP7NON), or "convenient" (DP4AST). While some parents perceived walking to be the easier mode compared to driving, others recognized the convenience of dropping their children off on their way to work in the car:

Ideally, I think it's great, it's a wonderful thing to be able to walk or cycle to school, but given the demands on parents these days, I think you often opt for what's most convenient and quickest, and allows you the time and supervision of the children. Often when you are working, even though there are opportunities to walk, time doesn't allow it and so you do what's best; so non-active modes of transport become the order of the day. (BP8NON)

Another NON AST parent concluded, "But again, it goes back to whether you want to do it. You always have to go back to that, if you can do it the easy way, I'm going to choose the easy way versus the best, healthy way to do it, I'm always going to choose the easy way, unfortunately" (RP8NON).

When prompted to provide suggestions about ways that schools could support AST, some of the NON AST parents from schools in low SES neighbourhoods stated that daycare centres or breakfasts programs would "make my life a lot easier" (DP7NON) and would make things "less complicated by possibly cutting back on the amount of time spent commuting in the morning and increasing the overall convenience of school travel" (DP8NON). Other factors that dictated the feasibility of either walking or driving to/from school were parents' work schedules. Both AST and NON AST parents explained how their work schedules dictated whether they were able to travel with their children to/from school since "the school world and the working world are not in sync" (RP4AST). One mother from a low SES neighbourhood school explained that it would be a challenge to walk to school because of her work commitments and that driving allows her to meet the demand of work and school routines:

[W]hen I leave home [in the morning] I take my son with me; I take him to my workplace because there's no point to get him to a babysitter for just an hour or a little bit over an hour. So he's at work with me until 8:30 am, and then I bring him to school and I finish work at 3 pm ... so I leave work at 8:30 am and reach back at about 8:50, so I would consider that my lunch. (TP10NON).

On the other hand, a mother from a high SES neighbourhood school, who walks to/from school, explained that "I have a flexible schedule ... I work from home, so it's easy ... Now if I had to work downtown and be there by 9 o'clock, I can see that crunch for time" (RP5AST). Those parents who worked part-time said that the days they did not work were usually the days they actively travelled to/from school with their kids. Work schedules, then, acted as a barrier to AST for NON AST parents particularly in terms of the time pressures associated with starting the work day.

However, work schedules were not the only factors that prevented AST. Participation in extra-curricular activities was also part of the broader trip chain to/from school for NON AST parents. Some of these participants reported that they needed to drive in order to get everyone (family members) to where they needed to be, and of course, get there on time. One mother from School D noted, ironically, that active travel was sometimes sacrificed in the mornings in order to get her son to school for his sporting activity:

[My son] really wanted to go to school early throughout September because they had cross country running, so the only way we could get to cross country running on time was to drive. So it's sort of an ironic thing that he turns down the opportunity to exercise in order to get to some collective exercise. (DP9NON)

Additionally, having to drop multiple children off at different schools also made AST challenging. A single parent from School R explained: "I have to get two other children to school directly after [my daughter] and then I have to go to work" (RP7NON). Therefore, most of the NON AST parents felt that they had no choice other than driving for their multiple school trips.

One interesting finding among these parents though was that the feasibility of walking as a mode choice differed in the AM and PM periods because of a change in the trip chain and work schedules described above. The majority of participants consistently used the same travel mode in the AM and PM periods. However, fluctuation in travel mode choice among typical active travelers was apparent on the trip to school, as these parents would sometimes drive their children to early morning practices or drop them off on their way to work. On the contrary, changes in travel mode choice among NON AST parents and their children most often affected the trip home from school in the afternoon (i.e., children who were driven sometimes walked home from school). This increase in active travel in the afternoon period seemed to reflect a time issue and/or parental availability due to work scheduling. Weekday mornings "seem to be worse" (DP9NON) because, as one mother from a low SES neighbourhood school explained, there is "so much to do, I mean, lunches, gathering up homework, getting people regimented and out the door. There's not a lot of sitting around" (DP9NON). Therefore, although time may be an issue in the morning for some, "after school it's not so much of an issue" (RP8NON).

#### Reinforcing Value

From the perspective of behavioural economics, decisions are also based on the reinforcing value (or reward) of engaging in a behaviour. Factors other than getting their child to/from school, on time and conveniently, reinforced parental decisions about mode choice. Both AST and NON AST parents explained how their decision often depended on the weather. While for one driver "there's no way" (RP8NON) she would choose to walk her children to/from school in the rain or snow, for some AST parents, such extreme elements made walking the preferred mode choice:

I mean, especially in the winter, I mean, I know people say they don't walk because of the weather, [but] I walk because of the weather in that for me to clear the driveway, clear the car, and get it out for such a short distance, it's silly, so we just walk. (RP2AST)

While parents who typically walked would choose to drive in what they perceived as 'bad weather', NON AST parents said that they would sometimes choose to walk on days when it was nice outside. One NON AST parent commented, "But if it's a beautiful day like summer or even fall, spring, even winter, if it's a nice day then we just walk" (BP9NON). Conversely, AST parents said that they would choose to drive if it's "raining very heavy" (DP1AST) or "it's freezing cold" (BP4AST). Therefore, parent's perceptions of 'nice weather' contributed to the overall appeal of walking to/from school. On the other hand, the rain and cold sometimes made walking unpleasant for these parents, and therefore a less desirable travel mode choice.

Parents of children attending high SES schools considered themselves "lucky" (BP4AST) to have the opportunity to walk to/from school with their children because of their living and working situations (e.g., walkable streets, flexible work times). They claimed that walking was the best mode if circumstances allowed for it to occur. Furthermore, these AST parents were the only participants who said that they walked as part of an overall "active healthy lifestyle" (BP2AST). One parent commented that "We're just active, there's no way that we will get into the vehicle and drive somewhere if we can walk or bike" (RP1AST). Although AST was part of an active healthy lifestyle for these parents who typically did walk to/from school, overwhelmingly, parents across all SES and built environments did not perceive the trip to/from school as an important opportunity for physical activity. One parent from a high SES neighbourhood school explained that actively travelling to/from school was not important for her family because they were already very active:

Well, the advantages of walking [to/from school] are health reasons, obviously - your activity. But that's not really a concern for us because we all do a lot of physical activity. For some families it's good because that may be the only physical activity their kids get, or even they get, but for us it's not a concern. (BP10NON)

The walk to/from school was also "not that big a deal" (BP4AST) because for some parents "it really isn't that long of a haul from our house to school" (DP1AST). Another parent from School R commented that "three minutes up the hill is really not what I would consider physical activity; it's just getting you outside" (RP4AST).

Only NON AST parents from School R (high SES, looping streets) discussed traffic (noise) and road safety as factors influencing travel mode choice. Most of these parents were concerned with the one major road bordering the school, and that influenced their decision to drive because of the "volume of traffic and getting across the street [safely]" (RP9NON). Another mother described the unpleasantness and negative impact of traffic noise:

[The street] is so busy; by the time you get home you're just stressed out because it's such a busy avenue ... It's just loud. If it [the walk] was into these little streets, I think that would be very enjoyable but the fact that I have to go through [the main street] to get to my house is just pretty loud, it's not like an enjoyable walk at all. (RP8NON)

In summary, many factors influenced the relative reinforcing value of using active or non-active modes for escorting children to/from school. NON AST parents from the high SES schools cited the weather as moderating mode choice. All NON AST parents said they might, sometimes choose to walk on days when the weather was 'nice'. AST parents from the high SES schools tended to endorse the benefits of walking to/from school as part of an overall active lifestyle (although the benefits were thought to be marginal when compared against other pre-existing forms of family physical activity) and the best mode choice if circumstances would allow. Traffic concerns reinforced parents' decisions to drive at the inner suburban schools.

## Discussion

Although the frameworks in the current AST literature [4,6,8] provide researchers with an intricate picture of the complex interplay of factors associated with school travel mode, they were not developed to articulate how these decisions are made. Our findings suggest that different factors influence travel mode choice at different stages of the decision-making process. This process is also not uniform for parents. Rather, some variation is evident on the basis of school location in terms of built environment characteristics and socioeconomic status. Traffic concerns were heightened in the inner suburban locations while parents of children from schools in the lower SES neighbourhoods reported less time available to walk with their child to/from school.

The decision making process with this group of parents involved two decisions: 1) decisions primarily concerned with escorting or independent travel, which appear to be primarily influenced by safety issues (e.g., traffic, strangers); and 2) decisions about the behavioural cost of walking versus driving which was largely conceptualized in terms of the time each took. First, parental concerns about traffic/road safety and stranger danger are not new to the school travel literature. Having 'concerns about traffic safety' is consistently reported as a barrier to AST [25-27] and increasingly parental concerns about personal safety have also been shown in the literature to be associated with decreased use of active modes for school travel (e.g., [17,28]). These fears are heightened if children commute to school independently [17]. In one qualitative study by Ahlport et al. [11], parents described their anxiety about letting their children "travel solo" because they would not know if they had arrived at school safely. Importantly, our study is illustrating that such factors extend across a multi-stage decision making process. Safety affects escorting, but might have less influence with regard to the choice of transport mode, once the escort decision has been taken.

Sirard and Slater [4] suggest that there needs to be a better understanding of parents' perceptions of their child's ability to navigate their physical and social environments. Both AST and NON AST parents expressed that they were uncomfortable with allowing their children to travel alone to/from school because they perceived that their children lacked the maturity and skill set needed to travel alone safely. Similar to other studies (e.g., [29]), we found that positive perceptions of neighbourhood social trust and cohesion moderated these fears among the parents in this study. The lack of safety skills necessary to walk to/from school have been associated with non-active forms of transportation in previous research [30]. Greves et al. [14] found that parents' perception of their school-aged child's 'immature judgment' (e.g., ability to follow traffic rules) was a barrier to AST. Johnston et al. [31] assessed a Walking School Bus initiative incorporating pedestrian safety skill instruction for inner public school children in Seattle. Using a pre-post test design, the researchers surveyed children's mode of transportation and also directly observed pedestrian safety behaviours. The initiative was deemed successful because the rates of children walking to school and children being driven to school increased and decreased, respectively. Furthermore, researchers found that there were slight improvements in observed measures of street-crossing safety, suggesting that teaching children the skills that are necessary for safely navigating streets may be effective in promoting AST. If parents perceive that their children have the necessary skill set to travel safely to/from school, then, this may reduce their apprehension about allowing their children to travel alone, thereby increasing the potential for AST.

Second, the behavioural cost of travel mode choice, largely influenced by parents' perceptions of time cost determined whether they escorted their child using AST or NON AST. Convenience was a central theme with respect to this decision. Surprisingly, the issue of convenience has received relatively little attention in the AST literature, although a recent study in the San Francisco Bay area reported that approximately 75% of parents driving their children less than 2 miles cited convenience as a reason [32]. In the current study, convenience was important for both AST and NON AST travel decisions. Factors influencing the time and convenience associated with school travel, such as work schedules, distance from school and activity trip chains, played a role in parents' decision making process.

A negative relationship between distance and AST is consistently reported in the literature [4]. Similarly, we found that both AST and NON parents explained that they walked and/or drove because of their proximity and distance to school, respectively. Trip chains that included travel to/from school also influenced the convenience of walking or driving. There is an emerging understanding of the interplay between parental work obligations, commuting, and school travel. In their qualitative studies, Ahlport et al. [11] and Greves et al. [14] reported that inflexible work schedules often prevented North Carolina and Seattle parents from walking with their children to school. Other studies have reported an association between children being driven to school and their parents' car journey to work [28,30,33,34]. Additionally, parents in Ahlport et al.'s [11] qualitative study described the convenience associated with driving their children to school on their way to work. Related issues of parental convenience and time constraints are increasingly being recognised as central reasons for why parents drive their children to school [32].

Conceptualizing the decision making process underpinning school travel mode choice in terms of two decisions has important implications for research and practice. In terms of research, studies typically associate correlates with the school travel mode choice only. Apart from distance, there is generally inconsistency in whether variables are positively, negatively, or not associated at all with travel mode (see [4]). Such inconsistency may be due to a failure to link the appropriate correlates with the proper decision within the overall decision making process. Specifically, safety issues appear to be more important for the escort decision but less so for the decision about the actual transport mode. Micro-level urban form features reflecting walkability (e.g., presence of sidewalks, intersections, density) may be integral to perceptions of safety. However, correlates reflecting time (e.g., distance) and convenience (e.g., trip-chain considerations) become more influential with regards to the final mode choice. Designing school neighbourhoods to reduce traffic around schools may ease parents' fears around independent travel; however, flexible work hours may play a more significant role on travel mode choice. Quantitative studies modelling the influence of different factors on school travel mode choice should consider accounting for these possibilities: there are some recent examples in the literature (e.g., [35-37]). In particular, Yarlagadda and Srinivasan [37] highlight the need to account for the likelihood that parents have a strong desire or need to chauffer their children to school. Our study reinforces the need to consider multiple household interactions and social relations, as well as activity-travel patterns when examining school transport.

Importantly, we need to recognize the broader physical and social environments within which both decisions are made. The design of neighborhoods, and where work takes place in relation to the home all influence where a household places the school trip and corresponding mode choice within the daily pattern of activities. The 'perception of convenience' emerges from within a complex web of urban development, political, and planning processes, and consumer choices, that collectively produce an allocation of individuals to neighbourhoods, jobs, and ultimately schools. Because SES can influence the built environment options available to individuals and households (e.g., the process of residential self-selection), and limit the options available to others, these factors must continue to be considered in order to understand how parents engage with the two-stage decision process described in this study. Social relations such as gender (e.g., who is more likely to travel to school with children), family structure (e.g., single or two parent households) and ethnicity (e.g., recently arrived immigrants who cannot access higher-paying jobs and are confined in their choices of neighbourhood schools or have limited daycare options) must also be considered in future studies of AST travel mode choice.

In terms of practice, this research suggests that interventions might be developed to more explicitly address escort decisions, and issues of convenience. Maintaining communication with their children by phone and having their children travel with friends and/or knowing other people in the neighbourhoods may help alleviate parents' concerns about independent travel. As noted by McDonald and Aalborg [32], offering non-infrastructure programs that provide adult supervision, such as Walking School Bus Schemes, may be a more powerful strategy to alleviate safety concerns while potentially reducing the parental time costs of escorting the child, than capital projects targeting changes to the built environment. However, we believe that such provisions may not be enough. There is little evidence that WSB schemes are effective and they may be difficult to sustain [38]. Accordingly, interventions should also be directed at the issue of convenience - an issue which is not commonly considered within intervention work such as Safe Routes to School Programs [32]. Behavioral economics provides an important perspective on how such interventions might be framed - can the behavioural cost and/or reinforcing value of mode choice alternatives be manipulated to change travel behaviour? What interventions can be developed that make driving the least convenient option (e.g., limited parking or "no vehicle zones" around school areas)? Or make walking more convenient (e.g., providing earlier school yard supervision; flexible school start/end times, and working schedules; availability of breakfast and/or childcare programming at schools)? How can decisions to walk to school be reinforced? Such considerations should be contextualized within broader policy initiatives that promote child and youth-friendly transport and land use planning (see [39]).

At this point, it is important to consider the limitations of this study and the transferability of the findings. First, parents who typically escorted their children to/from school were invited to participate in this study, and therefore we cannot conclude whether factors influencing travel decisions are similar for those parents who do not escort their children. The qualitative nature of this study does not lend itself to generalizing our findings across all school locations in a variety of cities and countries. However, by providing a thorough description of the research context, participants and findings, we believe that readers can evaluate the transferability of the findings to their own locations. Second, eight NON AST parents were recruited who did not live within the school catchment area (> 1.6 kms from school). Distance was the central factor influencing mode choice for these parents but they provided valuable insight into their travel experiences to/from school. There were certainly several notable strengths of this study. The sampling framework and sample size allowed for cross-site comparisons of factors influencing mode choice among parents from different SES backgrounds, whose children attended schools located in four different areas across Toronto. Additionally, our use of interpreters allowed us to capture the inclusion of parents who are traditionally excluded in research studies.

Overall, while the findings of this study reaffirm the role of many of the correlates reported in the AST literature, it provides new empirically-based findings to support further study of AST in terms of escort decisions and travel mode choice decisions. Notably, the trip to/from school, as a potential source of physical activity for their child(ren), was not important to the decision making process of the parents in this study. Rather, travel to/from school involves a two-step parental decision-making process and these choices are influenced by related but different factors. While escort decisions are dictated by road/traffic and personal safety concerns, the behavioural cost and reinforcing value of travel mode alternatives dictate mode choice. Our findings offer an important opportunity, then, to consider the links between school travel and the broader land use, and travel demand management strategies constructed by public and private institutions, with a view to re-organizing time and space within cities and regions to realign the consumption of housing, transport, and other activities, with a broader sustainability agenda. School travel is clearly linked to broader patterns of development (e.g., the decentralization of jobs for example) and economic specialization, as well as other systemic factors that we have mentioned above, that partially influence and sometimes entirely produce the location decisions of parents and households regarding housing and work, decisions that ultimately make "convenience" and "time use" the primary determinants of auto-oriented school transport. Our findings illustrate that you cannot solve the school travel 'problem', without considering the broader political, social and spatial planning context within which AST or NON AST is situated.

## Competing interests

The authors declare that they have no competing interests.

## Authors' contributions

GF and CF conceived of the study, and participated in its design and coordination and helped to draft the manuscript. RB participated in the design of the study, and drafted the manuscript. VR and FM participated in data collection and VR led data analysis. GF read the transcripts and validated the coding and analysis. All authors read and approved the final manuscript.

## Appendix 1 Sample Interview Questions

1. Can you tell me who makes the decision about how your child travels to school?

2. What factors influence the decision about how your child gets to school?

3. Describe a typical weekday morning - what activities do you and your family do before the journey to school? Do these activities influence your mode of transportation?

4. Where do you go after you drop your child off at school? Does this influence how you get to school in the morning?

5. What are alternative ways your child could get to school?

6. What are the advantages and disadvantages of these different ways?

Note: Repeated for the trip home from school.

## Appendix 2 Description of Transcript Codes

AST refers to walking to/from school. NON AST refers to driving to/from school. Participant quotes are identified using codes. The first letter of the code refers to the school (School T, B, R or D); the second letter of the code is "P" to identify the participant as a parent; the third part of the code is the participant number (1-10); the final part of the code characterizes participants as an AST or NON AST traveller. For example, code TP1AST refers to a parent from School T who walked to/from school.
